# Editorial: Recent advancements in the research models of infectious diseases

**DOI:** 10.3389/fcimb.2025.1731600

**Published:** 2025-11-07

**Authors:** Sam Ebenezer Rajadas, Christy Rosaline Nirmal, Azger Dusthackeer, Shakila Harshavardhan, Krupakar Parthasarathy

**Affiliations:** 1 Centre for Drug Discovery and Development, Sathyabama Institute of Science and Technology, Chennai, India; 2 Achira Labs Pvt., Ltd., Bengaluru, India; 3 ICMR-National Institute for Research in Tuberculosis, Chennai, India; 4 Department of Molecular Microbiology, School of Biotechnology, Madurai Kamaraj University, Madurai, India

**Keywords:** disease models, infectious diseases, research models, artificial intelligence in diagnosis, integrative disease modeling

Life on Earth is so pleasant till diseases and disorders strike. For long we believed that diseases were acts of God. It is no surprise that this was the time when people strongly supported the theory of spontaneous regeneration. The tables were turned upside down by two forefathers of microbiology, Louis Pasteur and Robert Koch. They were the first to experimentally use animal models, such as mice, rabbits, and chickens, especially in controlled settings, and exposed the pathogenicity of microbes and the experimental transmissibility of diseases. This led to the formulation of Koch’s Postulates in 1884 (Dewan and Harvill, 2024). Yet, the dynamic emergence of new pathogens and antimicrobial resistance necessitates continuous advancement in the study of infection biology, as it imposes a significant burden on global health and socioeconomic stability. Central to this endeavor is the development of robust, reproducible, and physiologically relevant models that exactly display host–pathogen interactions despite its complexity. Conventional models, including *in vitro* cell cultures, *in vivo* infection systems, and compartmental epidemiological frameworks, have long provided critical mechanistic insights. However, these approaches often fail to fully capture the spatial, temporal, and immunological characteristics (Swearengen, 2018). In recent years, an exciting wave of innovation has emerged, driven by advances in systems biology, high-throughput analytics, genetic engineering, and artificial intelligence, enlightening our understanding of the contagiousness, disease progression, and host immunological defense. This Research Topic, *Recent Advancements in the Research Models of Infectious Diseases*, was conceived to showcase current innovations that are transforming our understanding of infectious processes and to highlight the interplay between experimental systems, computational modeling, and translational applications with clinical relevance.

The articles in this Research Topic highlight a diverse range of infectious disease models. This includes mathematical and computational frameworks that are increasingly being leveraged to predict clinical outcomes, as demonstrated through studies that forecast mortality in COVID-19 patients (Xu et al.), and pneumonia-associated bloodstream infections (Zhou et al.) and even facilitating differential diagnosis (Peng et al.). These works highlight a growing recognition that predictive models, when anchored in patient-specific variables have significant potential to refine clinical decision-making and enhance the precision of infectious disease care.

At the experimental level, animal and novel nontraditional models are extending our capacity to study infection beyond conventional systems. Henriques et al. revisit the use of murine models to explore asymptomatic dengue infection, addressing both their utility and inherent limitations. Their review emphasizes the importance of refining animal models to better mirror subclinical infections that are often overlooked in spite of being epidemiologically significant (Henriques et al.). Complementing this perspective, another study explores fecal shedding patterns in SARS-CoV-2-infected rhesus macaques, providing valuable insight into viral persistence and host immune dynamics—an area with direct implications for understanding viral transmission routes and long-term pathogenesis (Böszörményi et al.).

Advances in diagnostics and biomarker-driven modeling was also featured prominently in this Research Topic. Li et al. present a stratified urine lipoarabinomannan assay for tuberculosis diagnosis, establishing a compelling link between host lymphocyte counts and pathogen-derived antigen detection. This study demonstrates how insights into the host–pathogen interface can enhance diagnostic sensitivity, particularly in immunocompromised populations where conventional methods often underperform (Li et al.). Expanding the modeling framework to an ecological scale, Zhao et al. used a MaxEnt-based approach to predict high-risk zones for avian influenza A (H7N9) infection, integrating environmental and epidemiological data to understand surveillance and preparedness strategies (Yang et al.). Additionally, the Research Topic underscores the emerging role of genomic insights, particularly those enabled by CRISPR-Cas systems, in simulating disease processes and driving novel therapeutic innovations (Al-Ouqaili et al.).

Multi-omics integration is becoming indispensable for unraveling the multilayered complexity of host responses. By merging transcriptomic, immunomic, and metabolomic data, researchers can construct network models that reveal hidden regulatory nodes and dynamic feedback loops during infection. Parallel to this, organoid and organ-on-chip systems are offering physiologically relevant microenvironments that bridge the gap between cell culture and whole-animal models (Barrila et al., 2018). These microphysiological systems enable detailed interrogation of tissue-specific infection processes while allowing manipulation of the mechanical, chemical, and immune microgradients that shape disease outcomes (Schmidiger and Portevin; Nangpal et al.).

Equally transformative are developments in computational and hybrid modeling. Artificial intelligence and machine-learning approaches, particularly, those constrained by biological or physical priors are accelerating our ability to simulate infection dynamics, predict drug responses, and identify emergent properties within complex datasets (Theodosiou and Read, 2023). Reaction diffusion and spatially explicit models are enriching our understanding of how infections propagate across tissues or populations, while phylodynamic frameworks integrating genomic and temporal data are reconstructing the invisible transmission networks that underlie epidemics (Waddel et al., 2025; Zhang and Wang, 2025). As these approaches evolve, the synergy between data-driven and mechanistic modeling will become highly significant to both fundamental and translational infection research.

Despite these advancements, challenges persist in integrating the immunological and multi-omics clinical datasets, which are prone to variability, missingness, and overfitting. The opacity of some artificial intelligent models and their algorithms can hinder clinical trust and mechanistic interpretation, thus warrants extensive validation along with ethical and biosafety considerations.

## Conclusion

Infectious disease modeling is no longer confined to isolated experimental or computational silos; it is now a multidisciplinary enterprise that thrives at the intersection of biology, data science, engineering, and clinical medicine. The most impactful studies are those that embrace repeated cycles of modeling, prediction, validation, and refinement, closing the gap between theory and experiment. As we move forward, integrating these approaches is essential to accelerate discovery, enhance preparedness for unprecedented infectious threats, improve diagnostics, therapeutics, and prevention strategies. We extend our sincere appreciation to all authors, reviewers, and editorial staff whose efforts made this Research Topic possible.
